# Supplementary material for: MetaRibo-Seq measures translation in microbiomes
Source: Nat Commun. 2020 Jun 29;11:3268. doi: 10.1038/s41467-020-17081-z (PMC7324362; doi:10.1038/s41467-020-17081-z)
Supplement: Supplementary file 10 — Supplementary Data 7 [file 41467_2020_17081_MOESM10_ESM.zip › File2/Confidence_High_Taxonomy/63884_out.krona.html]

Javascript must be enabled to view this page.

members
magnitude
magnitudeUnassigned
count
unassigned
taxon
rank

63884\_out

3

superkingdom
3
2

3
phylum
1239

class
3
186801

186802
3
order


SRS017916\_contig\_number\_14446
1898207
species
1

family
1
31979

1485
1
genus

1
species
2293014

SRS055982\_contig\_number\_contig-100\_6272.96126

1950837

SRS1054691\_contig\_number\_17556
species
1
